# Efficacy and Safety of Intravenous Ferric Derisomaltose for Chemotherapy-Induced Anemia: A Single-Center Retrospective Study in Japan

**DOI:** 10.7759/cureus.95349

**Published:** 2025-10-24

**Authors:** Junya Kamibayashi, Ryuji Kawaguchi, Yosuke Fukui, Naoki Kawahara, Kana Iwai, Yuki Yamada, Fuminori Kimura

**Affiliations:** 1 Obstetrics and Gynecology, Nara Medical University, Kashihara, JPN

**Keywords:** chemotherapy-induced anemia, ferric derisomaltose, intravenous, red blood cells, transfusion

## Abstract

Introduction

Chemotherapy-induced anemia (CIA) is a frequent complication in gynecologic cancers. In this study, we evaluated the effectiveness and safety of ferric derisomaltose (FDI), a high-dose intravenous (IV) iron formulation, for treating patients with CIA.

Methods

In this retrospective study, we analyzed patients with gynecologic cancers and CIA. A total of 29 patients with hemoglobin (Hb) levels <9.0 g/dL, or who were anticipated to fall below this threshold during chemotherapy, received a single 1,000 mg dose of FDI between 2023 and 2024. As a historical control group, 51 patients treated between 2020 and 2021 who did not receive IV iron were included. The primary outcome was the change in Hb level on day 21. Secondary outcomes included red blood cell (RBC) transfusion rates and the incidence of chemotherapy discontinuation, delay, or dose reduction.

Results

The baseline Hb levels were 7.6 ± 0.4 g/dL in the FDI group and 7.9 ± 0.4 g/dL in the control group. By day 21, the mean Hb level had increased to 9.4 ± 1.0 g/dL in the FDI group, whereas it declined to 7.4 ± 0.7 g/dL in the control group (p < 0.001). Significantly fewer patients required RBC transfusions in the FDI group than in the control group (two [6.9%] and 17 patients [33.3%], respectively; p = 0.007). Chemotherapy modifications occurred in five patients (17.2%) in the FDI group versus 11 patients (21.6%) in the control group (p = 0.642).

Conclusion

FDI significantly increased the Hb levels and reduced transfusion requirements in patients with gynecologic cancers and CIA. It was well tolerated and may represent a valuable alternative therapeutic option for managing CIA.

## Introduction

Chemotherapy-induced anemia (CIA), primarily caused by myelosuppression during chemotherapy, is among the most common adverse events associated with anticancer treatments [1]. It is estimated that 30-90% of patients with solid tumors and 26-85% of those with gynecologic cancers develop anemia during chemotherapy [1-3].

Anemia during chemotherapy impairs patients’ quality of life and necessitates dose reductions or discontinuation of anticancer therapy, thereby potentially compromising treatment efficacy. Therefore, effective management of CIA can have a significant impact on patient prognosis [4].

According to the National Comprehensive Cancer Network (NCCN) guidelines version 1.2025, an evaluation for anemia-related symptoms is recommended when hemoglobin (Hb) levels fall to ≤11.0 g/dL or decrease by ≥2 g/dL from baseline during chemotherapy [5]. If patients exhibit symptoms associated with anemia, red blood cell (RBC) transfusion is recommended [4]. In asymptomatic patients, iron deficiency should be evaluated; if confirmed, oral or intravenous (IV) supplementation should be considered [4].

The criteria for iron deficiency differ across guidelines. According to NCCN criteria, absolute iron deficiency is defined as transferrin saturation (TSAT) <20% and serum ferritin <30 ng/mL, whereas functional iron deficiency is defined as TSAT <50% and ferritin between 30 and 500 ng/mL [5]. For patients with absolute iron deficiency, iron supplementation (IV or oral) is advised, whereas in cases of functional iron deficiency, combination therapy with IV iron and erythropoiesis-stimulating agents (ESAs) is recommended.

The European Society for Medical Oncology (ESMO) guidelines [6] similarly recommend IV iron (1,000 mg) for patients with Hb 10.0-11.0 g/dL and TSAT <20% or ferritin <100 ng/mL. For patients with Hb <10.0 g/dL and absolute iron deficiency, IV iron is the preferred treatment, whereas in functional iron deficiency, ESAs combined with IV iron are advised.

A retrospective cohort study involving 25,018 patients with cancer reported that 11,019 developed anemia within six months of initiating treatment [7]. Among these, 4,318 patients (39%) received anemia treatment. RBC transfusion was the most common intervention (3,528 patients, 32%), while oral iron (1,279 patients, 12%) and IV iron therapy (97 patients, 1%) were less frequently used.

Similarly, in a single-center retrospective cohort study [8] of 939 (44%) patients with gynecologic cancers, 625 (67%) developed anemia (Hb ≤ 11 g/dL) within six months of treatment. Of these, 260 patients (42%) received anemia treatment, comprising RBC transfusion (205 patients, 22%), oral iron supplementation (57 patients, 6%), and IV iron therapy (eight patients, 0.8%).

These findings underscore a persistent gap in adherence to NCCN guidelines, with low rates of iron supplementation for CIA. In Japan, as in many other countries, severe CIA is primarily managed with RBC transfusion, whereas mild to moderate anemia is often deemed clinically insignificant and rarely treated with iron supplementation.

Ferric derisomaltose (FDI) is a high-dose IV iron formulation approved in Japan for treating iron deficiency anemia. The NCCN guidelines list FDI as one of the therapeutic options for CIA. However, to our knowledge, no reports on the use of FDI for CIA in Japan have been published. In this study, we aimed to retrospectively evaluate the efficacy of FDI in the treatment of CIA.

## Materials and methods

Study population

Inclusion Criteria

The FDI group included patients with gynecologic cancers who received chemotherapy and were treated with FDI for CIA at the Department of Obstetrics and Gynecology, Nara Medical University, Kashihara, Japan, between July 2023 and December 2024. FDI was administered to patients undergoing chemotherapy and whose Hb levels were <9.0 g/dL or who were expected to fall below this threshold. A single IV dose of 1,000 mg FDI was administered over 30 min.

The historical control group included patients with gynecologic cancers who received chemotherapy and whose Hb levels were <9.0 g/dL between January 2020 and March 2021, without the use of IV or oral iron supplementation in our institution. Both groups included patients who required RBC transfusions.

Exclusion Criteria

Patients who could not be assessed for Hb level on days 0, 7, 14, and 21 were excluded from both groups.

Sample Size

As all patients who met the inclusion criteria within the specified period were analyzed, the sample sizes were not calculated for either group.

This study was approved by the Institutional Review Board of Nara Medical University Hospital (approval number: 3733). The requirement for informed consent was waived in accordance with the opt-out approach. 

Clinical outcomes

Data on patient characteristics (age, body mass index, and type of gynecologic cancer), chemotherapy regimens, and hematological parameters were retrospectively collected from medical records. The efficacy of FDI in treating CIA, measured by improvement in anemia, reduction in RBC transfusion requirements, and prevention of chemotherapy discontinuation or dose modification, was evaluated through comparison with the historical control group. Adverse drug events were assessed using the Common Terminology Criteria for Adverse Events (CTCAE) version 5.0.

In the FDI group, day 0 was defined as the day of FDI administration. In the control group, day 0 was defined as the date of anemia diagnosis. Hb levels were assessed on days 0, 7, 14, and 21 in both groups. Hb values at each time point were measured and compared between the FDI and control groups.

Statistical analysis

All statistical analyses were performed using IBM SPSS Statistics, version 24.0 (IBM Corp., Armonk, NY). Baseline characteristics and hematological parameters between the FDI and control groups were compared using the Mann-Whitney U test and Fisher’s exact test, as appropriate.

Changes in Hb levels over time were compared between the two groups using two-way analysis of variance followed by Bonferroni correction. A p-value of <0.05 was considered statistically significant.

## Results

Patient characteristics

The patient characteristics of the 29 patients in the FDI group and 51 patients in the control group (total n = 80) are summarized in Table 1. There were no significant differences between the groups in terms of mean age or mean body mass index. The distribution of gynecologic cancer types was similar: in the FDI group, there were six patients with cervical cancer, three with endometrial cancer, 18 with ovarian cancer (including primary peritoneal and fallopian tube cancers), and two with other types. In the control group, 17 had cervical cancer, eight had endometrial cancer, 24 had ovarian cancer, and two had other cancer types.

**Table 1:** Patient characteristics in the FDI and control groups Statistical methods are the Mann-Whitney U test and Fisher’s exact test. A p-value of <0.05 was considered statistically significant. The values are presented as means ± standard deviation. BMI, body mass index; FDI, ferric derisomaltose; MCV, mean corpuscular volume

Characteristics	FDI group	Control group	p-value
Cases (number)	29	51	-
Age (years)	61.4 ± 15.2	61.9 ± 14.2	0.78
BMI (kg/m^2^)	22.4 ± 3.9	22.2 ± 3.7	0.725
Type of cancer (number, %)			
cervical cancer	6 (20.7%)	17 (33.3%)	0.469
endometrial cancer	3 (10.3%)	8 (15.7%)	-
ovarian cancer/tubal cancer/peritoneal cancer	18 (62.1%)	24 (47.1%)	-
others (vulva cancer/uterine sarcoma)	2 (6.9%)	2 (3.9%)	-
Chemotherapy (number, %)			
First-line chemotherapy	15 (51.7%)	24 (47.1%)	0.433
Second-line chemotherapy	14 (48.3%)	27 (52.9%)	-
Prior chemotherapy regimens (number, %)			
0	15 (51.7%)	24 (47.1%)	0.92
1	4 (13.8%)	8 (15.7%)	-
≥2	10 (34.5%)	19 (37.3%)	-
Laboratory data			
Hemoglobin (g/dL)	7.6 ± 0.6	7.9 ± 0.5	0.021
MCV (fL)	89.8 ± 8.3	88.5 ± 4.3	0.065
White blood cell count (/µL)	3,731 ± 1,653	3,998 ± 4,228	0.746
Neutrophil count (/µL)	2,289 ± 1,884	2,580 ± 3,679	0.692
Platelet count (104/µL)	15.9 ± 4.7	15.7 ± 3.6	0.928

Regarding treatment lines, 15 patients in the FDI group and 24 patients in the control group received first-line chemotherapy, whereas 14 and 27 patients, respectively, received second-line chemotherapy. As for prior chemotherapy exposure, four patients in the FDI group had received one regimen and 10 had received two or more; in the control group, eight had received one regimen and 19 had received two or more. 

At the time of CIA diagnosis during chemotherapy, the mean Hb level was 7.6 ± 0.6 g/dL in the FDI group and 7.9 ± 0.5 g/dL in the control group, with a statistically significant difference (p = 0.021). There were no significant differences between the groups in the mean corpuscular volume, white blood cell count, neutrophil count, or platelet count. 

Chemotherapy regimens

Chemotherapy regimens at the time of CIA diagnosis are summarized in Table 2. In the FDI group, 21 patients (72.4%) were treated with combination therapy and eight patients (27.6%) received monotherapy. In the control group, combination therapy was used in 33 patients (64.7%) and monotherapy in 18 patients (35.2%).

**Table 2:** Chemotherapy regimens at the time of CIA diagnosis in the FDI and control groups CIA, chemotherapy-induced anemia; FDI, ferric derisomaltose

Group	Number
FDI group	
Combination therapy	21 (72.4%)
Carboplatin/paclitaxel (TC)	12 (41.4%)
Docetaxel/gemcitabine	2 (6.9%)
TC/pembrolizumab	1 (3.4%)
TC/bevacizumab	1 (3.4%)
TC/bevacizumab/olaparib	1 (3.4%)
TC/pembrolizumab/bevacizumab	1 (3.4%)
Carboplatin/docetaxel	1 (3.4%)
Liposomal doxorubicin/bevacizumab	1 (3.4%)
Bleomycin/etoposide/cisplatin	1 (3.4%)
Bevacizumab/olaparib	1 (3.4%)
Monotherapy	8 (27.6%)
Niraparib	3 (10.3%)
Liposomal doxorubicin	3 (10.3%)
Cisplatin	1 (3.4%)
Control group	
Combination therapy	33 (64.7%)
Carboplatin/paclitaxel (TC)	20 (39.2%)
TC/bevacizumab	4 (7.8%)
TC/pembrolizumab	3 (5.9%)
Cisplatin/doxorubicin	2 (3.9%)
Cisplatin/irinotecan	1 (2.0%)
Bleomycin/etoposide/cisplatin	1 (2.0%)
Carboplatin/etoposide	1 (2.0%)
Lenvatinib/pembrolizumab	1 (2.0%)
Monotherapy	18 (35.2%)
Nedaplatin	4 (7.8%)
Liposomal doxorubicin	4 (7.8%)
Cisplatin	3 (5.9%)
Nogitecan	2 (3.9%)
Paclitaxel	1 (2.0%)
Irinotecan	1 (2.0%)
Gemcitabine	1 (2.0%)
Niraparib	1 (2.0%)
Olaparib	1 (2.0%)

Hb changes

**Figure 1:**
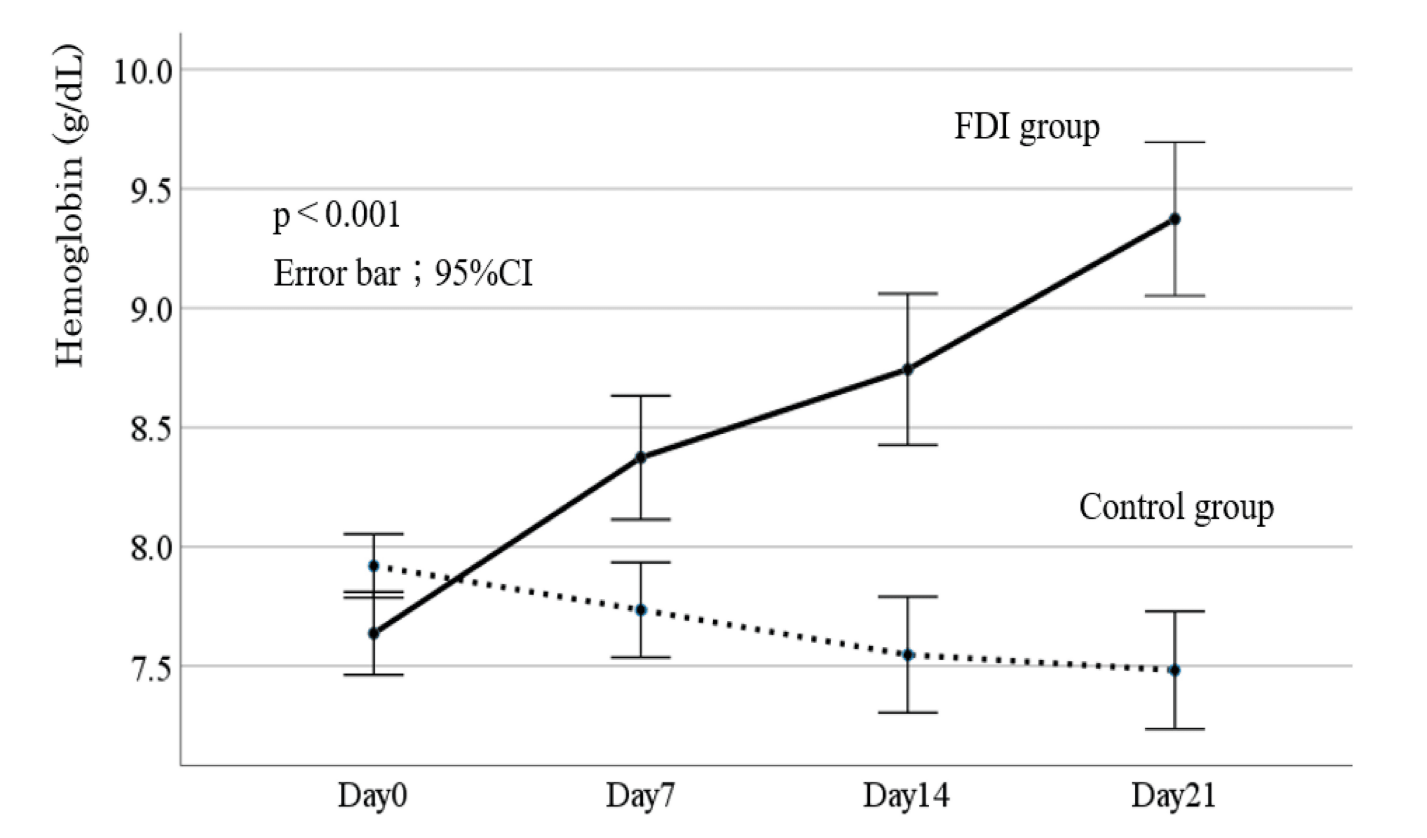
Comparison of changes in hemoglobin levels between the FDI and control groups Statistical methods are a two-way analysis of variance followed by the Bonferroni correction. A p-value of <0.05 was considered statistically significant. FDI, ferric derisomaltose

**Figure 2:**
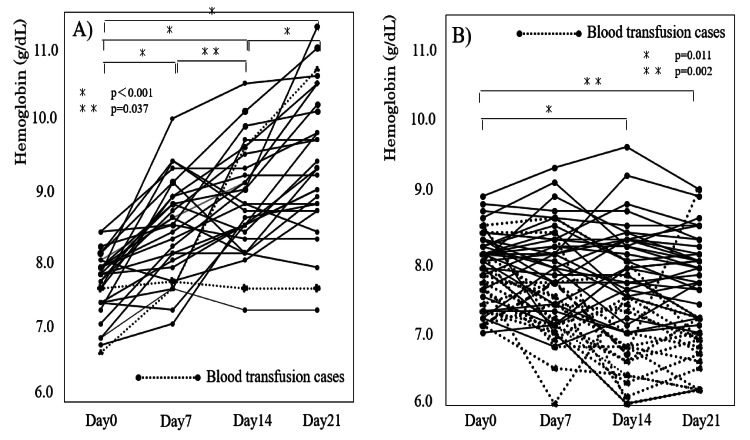
Changes in hemoglobin levels in the FDI (A) and control groups (B) Statistical methods are a two-way analysis of variance followed by the Bonferroni correction. A p-value of <0.05 was considered statistically significant. FDI, ferric derisomaltose

Figure 1 presents the comparison of Hb changes between the FDI and control groups. From days 0 to 21, Hb levels increased in the FDI group but decreased in the control group, a difference that was significant (p < 0.001). Figure 2 shows the changes in Hb levels over time for all patients in each group. In the FDI group, the mean Hb level increased from 7.6 ± 0.4 g/dL on day 0 to 8.4 ± 0.7 g/dL on day 7, 8.8 ± 0.7 g/dL on day 14, and 9.4 ± 1.0 g/dL on day 21. By contrast, in the control group, the mean Hb level declined from 7.9 ± 0.4 g/dL on day 0 to 7.7 ± 0.6 g/dL on day 7, 7.5 ± 0.8 g/dL on day 14, and 7.4 ± 0.7 g/dL on day 21. No hypersensitivity reactions were observed in the FDI group.

Transfusion and chemotherapy modifications

Table 3 shows the RBC transfusion rates and the incidence of chemotherapy discontinuation or dose reduction within 21 days of CIA diagnosis. RBC transfusion was required in two patients (6.9%) in the FDI group and 17 patients (33.3%) in the control group, representing a significant difference (p = 0.007). Discontinuation or dose reduction of anticancer drugs was observed for five patients (17.2%) in the FDI group and 11 patients (21.6%) in the control group, with no significant difference (p = 0.642).

**Table 3:** RBC transfusion and discontinuation/reduction of anticancer drugs within three weeks of diagnosis of CIA Statistical methods are the Mann-Whitney U test and Fisher’s exact test. A p-value of <0.05 was considered statistically significant. RBC, red blood cell; CIA, chemotherapy-induced anemia; FDI, ferric derisomaltose *reduction, three cases; discontinuation, one case; reduction and discontinuation, one case. **reduction, two cases; discontinuation, one case; reduction and discontinuation, eight cases.

Variables	FDI group (29 cases)	Control group (51 cases)	p-value
RBC transfusion	2 (6.9%)	17 (33.3%)	0.007
Discontinuation/dose reduction of anticancer drugs	5* (17.2%)	11** (21.6%)	0.642

## Discussion

To our knowledge, this retrospective study is the first to evaluate the efficacy of FDI for CIA in Japan. Our findings demonstrate that a single 1,000 mg IV dose of FDI effectively corrected Hb levels in patients with anemia undergoing chemotherapy, without the use of ESAs.

According to the NCCN guidelines, anemia during chemotherapy is defined as an Hb level of ≤11 g/dL or a ≥2 g/dL decrease from baseline. Hematologic toxicity owing to chemotherapy is typically graded using the CTCAE [9], with grade 3 anemia, defined as an Hb level <8.0 g/dL, often necessitating chemotherapy dose reduction or discontinuation. For patients experiencing rapid Hb decline, comorbid conditions (such as cardiac, pulmonary, or cerebrovascular disease), or anemia-related symptoms (including tachycardia, chest pain, dizziness, or dyspnea), RBC transfusion is recommended. In the absence of transfusion, patients with CIA should be evaluated for iron deficiency [5].

Although ESAs are associated with benefits, such as improved Hb levels, reduced transfusion needs, and alleviation of anemia-related symptoms, they carry risks, including thromboembolic events [6,10]. Recent meta-analyses have raised concerns about the negative impact of ESAs on survival, suggesting that excessive Hb elevation and increased thrombotic risk may contribute to poorer outcomes [11]. In Japan, the use of ESAs is not approved for CIA due to these concerns. Therefore, based on our findings, high-dose iron supplement therapy, such as FDI, appears to be an important therapeutic strategy for patients with CIA.

Despite clear recommendations from the NCCN and ESMO, compliance with anemia management guidelines remains low. A retrospective cohort study involving 25,018 patients with solid tumors showed that among 11,019 (44%) patients who developed CIA within six months of diagnosis, only 1,742 (7%) underwent iron status testing, and only 97 (0.4%) received IV iron therapy [6]. Similarly, among 625 (2%) patients with CIA secondary to gynecologic cancer, iron status was assessed in only 80 (0.3%), and only eight (0.03%) received IV iron therapy [8]. In particular, the role of high-dose IV iron formulations warrants further prospective evaluation. These findings underscore the urgent need to improve the implementation of CIA management protocols.

At our institution, RBC transfusion has been the mainstay of CIA treatment. However, this study shows that high-dose IV iron with FDI is an effective alternative. In a Japanese survey on CIA, conducted by the Japan Society of Clinical Oncology and the Japanese Society of Transfusion Medicine and Cell Therapy, 7.5% of patients undergoing chemotherapy received RBC transfusions, with an average of 5.9 units per patient [12]. The survey highlighted the need for alternatives, such as ESAs, to enhance quality of life, but did not mention iron evaluation or iron therapy. Notably, ESA therapy for CIA is not currently covered by insurance in Japan.

Several clinical trials have reported that IV iron monotherapy improves the Hb levels and reduces the need for transfusions in patients with CIA [13-16]. Based on these findings, IV iron, either alone or as part of combination therapy, may offer advantages over ESA therapy. Our results suggest that FDI can reduce reliance on RBC transfusions, which are associated with risks, such as transfusion-related complications and alloimmunization.

The PROFOUND study, which evaluated the non-inferiority of FDI compared with oral iron in CIA, found that FDI was equally effective in elevating Hb levels and was better tolerated, with fewer adverse events, such as fatigue [17]. No treatment-related adverse events associated with FDI were observed in this study. FDI became available in Japan in March 2023 as a high-dose IV iron product approved for the treatment of iron deficiency anemia.

FDI enables the administration of a single 1,000 mg dose, offering convenience and effectiveness in raising Hb levels and reducing transfusion dependence. In this study, 29 patients with gynecologic cancers and CIA were treated with FDI, among whom 27 (93.1%) avoided transfusions, with only two patients requiring them. Notably, our patients had lower baseline Hb levels (Hb < 8.0 g/dL or expected to drop below) than those in the PROFOUND study, where the inclusion criterion was Hb ≤12.0 g/dL [17]. It is possible that administering FDI at a higher Hb threshold (e.g., 9-12 g/dL) further decreased the need for transfusions in our cohort.

Limitations

First, this study utilized a historical control group that did not receive IV iron supplementation for CIA as a comparator. Consequently, potential differences in baseline characteristics, healthcare environments, and concomitant therapies between the historical and intervention groups may have introduced systematic bias that could affect the outcomes.

Second, the number of patients in the FDI group was relatively small (n = 29), and the retrospective design inherently limits the ability to establish causal relationships. To validate these findings, future large-scale, prospective, randomized controlled trials are warranted.

Third, we did not assess serum ferritin and TSAT levels in enrolled patients. As a result, it was not possible to determine whether patients had absolute or functional iron deficiency. However, given that most prior studies of CIA included patients with Hb levels ≤11.0-12.0 g/dL, and our cohort consisted of patients with even lower Hb values, it is reasonable to presume that a substantial proportion had absolute iron deficiency.

Fourth, the long-term safety of FDI administration in patients with CIA remains uncertain. Although FDI and other IV iron preparations have demonstrated short-term efficacy, their long-term safety profiles in oncology populations are not yet fully established and require further investigation.

Finally, we could not assess the adverse events in the control group because they were collected historically. Consequently, the incidence of adverse events could not be accurately compared between the FDI and control groups.

## Conclusions

Patients with CIA in Japan are traditionally managed with RBC transfusions and oral iron supplementation. However, RBC transfusions carry risks such as allergic reactions and increased medical costs, while oral iron supplements are poorly absorbed in patients with cancer. This is the first retrospective study in Japan to demonstrate the effectiveness of FDI in treating patients with gynecologic cancer and CIA. We demonstrated that administration of high-dose IV iron (FDI) in these patients with CIA associated with gynecologic cancers resulted in increased Hb levels and enabled the avoidance of blood transfusions. These results are expected to significantly influence treatment strategies in patients with gynecologic cancer and CIA.
